# Associations between inactivated COVID-19 vaccination status and timing and fertility and pregnancy outcomes following frozen-thawed embryo transfer: a prospective cohort study

**DOI:** 10.3389/fendo.2025.1587251

**Published:** 2025-06-26

**Authors:** Danmeng Liu, Lijuan Chen, He Cai, Hanying Zhou, Min Wang, Na Li, Xia Xue, Li Tian, Ben W. Mol, Wenhao Shi, Juanzi Shi

**Affiliations:** ^1^ Translational Medicine Center, Northwest Women’s and Children’s Hospital, Xi’an, Shaanxi, China; ^2^ Assisted Reproduction Center, Northwest Women’s and Children’s Hospital, Xi’an, Shaanxi, China; ^3^ Department of Obstetrics and Gynaecology, School of Clinical Sciences at Monash Health, Monash University, Melbourne, VIC, Australia; ^4^ Department of Obstetrics and Gynaecology, Amsterdam University Medical Centre, Amsterdam, Netherlands

**Keywords:** COVID-19, inactivated vaccine, *in vitro* fertilization, frozen-thawed embryo transfer, clinical pregnancy, live birth

## Abstract

**Objective:**

Limited evidence exists on the safety of inactivated COVID-19 vaccines and the optimal vaccination timing for women undergoing *in vitro* fertilization-frozen embryo transfer (IVF-FET). This study aims to examine the associations between inactivated COVID-19 vaccination status and timing and fertility and pregnancy outcomes following IVF-FET.

**Methods:**

This was a single-center prospective cohort study conducted from 1 May to 31 December 2021, with follow-up until 15 November 2022. We studied female patients aged 20 to 47 years undergoing IVF. Participants undergoing their first FET were included in this study. Information on maternal sociodemographic and health-related factors, COVID-19 vaccination status, the IVF-ET process, and outcomes was collected. Generalized linear models or generalized estimating equation models were used to evaluate the associations between vaccination and fertility and pregnancy outcomes. We controlled for maternal characteristics and cycle characteristics, including maternal age, BMI, parity, ovarian stimulation protocol, fertilization method, endometrial preparation, and other relevant factors.

**Results:**

A total of 2,733 eligible women were included, with 742 (27.1%) in the vaccinated group and 1,991 (72.9%) in the unvaccinated group. Among these women, 1,367 (50.0%) achieved a live birth. The incidences of clinical pregnancy and live birth were lower in the vaccinated group compared to the unvaccinated group (clinical pregnancy: 56.6% vs. 63.6%; adjusted RR: 0.92, 95% CI: 0.86, 0.98; live birth: 44.3% vs. 52.2%; adjusted RR: 0.89, 95% CI: 0.82, 0.98). These significant associations were more pronounced in women vaccinated before ovarian stimulation (clinical pregnancy: adjusted RR: 0.91, 95% CI: 0.84, 0.98; live birth: adjusted RR: 0.86, 95% CI: 0.78, 0.95), particularly in those with a ≤ 90-day interval between vaccination and ovarian stimulation. The effect size was similar but did not reach statistical significance in women vaccinated after ovarian stimulation and before FET (clinical pregnancy: adjusted RR: 0.92, 95% CI: 0.79, 1.07; live birth: adjusted RR: 0.93, 95% CI: 0.78, 1.12). No significant association was found with pregnancy outcomes.

**Conclusion:**

Inactivated COVID-19 vaccination may be associated with a modest reduction in IVF-FET success, particularly when administered before ovarian stimulation. However, a vaccination administered more than 90 days prior to ovarian stimulation may help mitigate these potential adverse effects.

## 1 Introduction

Since December 2019, the severe acute respiratory syndrome coronavirus 2 (SARS-CoV-2) has caused several waves of the coronavirus disease 2019 (COVID-19) pandemic, resulting in over 776 million confirmed cases and more than 6.9 million deaths worldwide as of 19 May 2024 (1, 2). COVID-19 can induce serious multi-organ dysfunction in human body and add an enormous disease burden (3). Give these impacts, COVID-19 vaccination has been highly recommended by the World Health Organization and national governments and has been administered globally to provide strong protection against serious illness, hospitalization, and death from COVID-19 (4, 5).

Data on vaccine safety when used before or during pregnancy were limited, particularly for women receiving *in vitro* fertilization (IVF) treatment (5, 6). Although existing observational studies have not identified any reproductive-specific safety concerns for women who have received SARS-CoV-2 vaccines during pregnancy or before assisted reproductive technology (ART) treatment (4, 7), the influence of vaccination early in pregnancy, optimal vaccination timing or time interval between vaccination and ART treatment, and long-term impact on female/male reproductive function and offspring’s health remain unclear (4, 6–8). Additionally, previous studies primarily focused on the mRNA vaccines (5, 9). Consequently, the uncertainty regarding the impact has led to low vaccination willingness among women undergoing ART treatment in China (7, 10), where inactivated vaccines are predominantly used and account for over 85% of total COVID-19 vaccinations (11).

Recent evidence has suggested that inactivated COVID-19 vaccination before pregnancy can protect the fetus by enhancing the fetal immune regulation when the mother is infected with SARS-CoV-2 during pregnancy (12). This highlights the effectiveness and protective effect of vaccination. Accordingly, for women planning to undergo or currently undergoing IVF treatment, there is an urgent need for more evidence on the safety of inactivated vaccines to dispel their concerns and to clarify the optimal timing of vaccination. This is crucial for establishing appropriate clinical recommendations for vaccination and ART treatment schedules.

Recent limited evidence from meta-analyses was conflicting. For instance, a recent study by Zhang et al. found that COVID-19 vaccination, particularly with inactivated vaccines, may reduce clinical pregnancy rates (8). However, another study by Chamani et al. did not find any significant impact of vaccination, including both inactivated and mRNA vaccines, on IVF outcomes (13). This inconsistency may be due to differences in the studies included and the specific vaccine types examined. Moreover, current studies have not provided meta-analysis data on the impact of COVID-19 vaccines on fresh or frozen embryo transfer outcomes.

For women undergoing IVF with fresh embryo transfer (ET), previous studies have suggested that inactivated COVID-19 vaccination may negatively impact oocyte maturation, fertilization rates, and pregnancy rates. The potential mechanisms involve elicited inflammatory responses that could interfere with folliculogenesis, leading to abnormal oocytes and fertilization (14, 15). Additionally, existing evidence has proposed that an interval of at least 61 days between the first dose of the vaccine and the initiation of fertilization treatment may be necessary to minimize potential adverse effects (15). However, for women undergoing frozen-thawed embryo transfer (FET), the relationship between vaccination status and reproductive outcomes remains controversial. Current evidence presents conflicting conclusions regarding ongoing pregnancy and live birth rates. Moreover, existing studies have not established an optimal vaccination timing relative to FET cycles. These knowledge gaps are primarily attributed to limitations in the existing research, including small sample sizes, significant heterogeneity among study populations, and considerable variability in study outcomes (9, 10, 16, 17).

Therefore, in light of the limitations of previous studies, the present prospective cohort study aimed to investigate the associations between inactivated COVID-19 vaccine inoculation and fertility and pregnancy outcomes among women undergoing FET, and to evaluate the proper vaccination timing for IVF treatment.

## 2 Materials and methods

### 2.1 Study design and participants

This prospective cohort study was conducted at the Assisted Reproduction Center of the Northwest Women’s and Children’s Hospital in China. Patients undergoing IVF treatment between 1 May and 31 December 2021 were registered on the day of oocyte retrieval. The inclusion criteria for the participants were as follows: 1) female patients aged 20–47 years old; 2) patients undergoing IVF or intracytoplasmic sperm injection (ICSI) treatment. After registration, participants were followed up until the investigated fertility or pregnancy outcomes occurred (the study ended on 15 November 2022).

According to the objective of the present study, we included women who underwent their first FET in a freeze-all cycle or a non-elective FET cycle subsequent to a fresh ET cycle. In the non-elective FET cycle, supernumerary embryos were used following a fresh ET cycle that did not achieve a live birth. Participants who met any of the following criteria were excluded: 1) did not complete follow-ups until the end of the study; 2) had three or more controlled ovarian stimulation (COS) cycles; 3) used donated sperm or donated/frozen/thawed oocytes; 4) underwent preimplantation genetic testing cycles; 5) received other types of COVID-19 vaccine or had invalid vaccine information; 6) were vaccinated after FET during follow-ups; 7) had SARS-CoV-2 infection before or during IVF-FET treatment, or during follow-ups; 8) had missing data during treatment or follow-ups.

### 2.2 Data collection

Participants’ registration and follow-ups were conducted at the Assisted Reproduction Center by trained physicians. During registration, information on maternal sociodemographic and health-related characteristics and SARS-CoV-2 infection history was collected via face-to-face interviews and entered into the electronic medical record system. Information on the IVF process and related health status was recorded in the electronic medical record system during treatment and follow-ups.

Detailed COVID-19 vaccination status was collected at registration and follow-ups using an online questionnaire. Information on vaccine type, vaccination date, number of doses, manufacturer name, and adverse reactions experienced after vaccination was collected. During the COVID-19 pandemic in China, every individual’s vaccination record is meticulously documented by the government. People can access accurate and detailed information about their vaccinations through their smartphone apps (Yi Ma Tong, Alipay, or WeChat), which are officially authorized to provide verified and detailed vaccination records. To ensure data accuracy, the participants were specifically instructed to access their immunization records through the local public health surveillance system using these apps. For patients who did not complete the online questionnaire or had incomplete data, we followed up on this information through telephone calls to ensure a sufficient questionnaire response rate.

The methods of the ovarian stimulation protocol, embryo vitrification and thawing procedures, endometrial preparation protocols, and frozen-thawed embryo transfer are described in the **Supplementary Materials**.

### 2.3 Exposure and outcome assessment

The exposure factor in the present study was COVID-19 vaccination status. Participants who had received COVID-19 vaccinations before FET were categorized as the vaccinated group, while those who had not received any vaccinations were categorized as the unvaccinated group. In addition to vaccination status, based on the timing of the first dose of vaccination relative to COS and FET, vaccinated women were further classified into two subgroups: those vaccinated before ovarian stimulation (before OS group) and those vaccinated after OS and before FET (after OS and before FET group). Furthermore, according to the time interval between OS and vaccination or between vaccination and FET, the women in the before OS group were subdivided into two groups: those vaccinated ≤ 90 days before OS and those vaccinated > 90 days before OS. Similarly, for the group vaccinated after OS and before FET, they were classified into two subgroups: those vaccinated ≤ 90 days before FET and those vaccinated > 90 days before FET (15, 18).

The primary outcomes of this study were clinical pregnancy and live birth per cycle. Clinical pregnancy was defined as the presence of an intrauterine gestational sac with or without a fetal heartbeat on ultrasonography during the first trimester. Live birth was defined as a viable infant delivered after a complete gestational period of 24 weeks or longer. The secondary outcomes included biochemical pregnancy, ongoing pregnancy, and pregnancy outcomes including neonatal birth weight, gestational age, preterm birth, low birth weight (LBW), and macrosomia. Biochemical pregnancy was assessed by a positive serum β-human chorionic gonadotropin (hCG) level 12 to 14 days after the embryo transfer. Ongoing pregnancy was defined as a clinical pregnancy that continued for at least 12 weeks. Birth weight was measured to the nearest 10 g with a baby scale within 1 hour after delivery. The child’s sex (male/female) was recorded after delivery. Gestational age at delivery was calculated according to the last menstrual period and was confirmed by ultrasound scans. Preterm birth was defined as delivery after 24 weeks but less than 37 weeks of gestation. According to the WHO, LBW was defined as birth weight less than 2,500 g (19) and macrosomia was defined as a birth weight of no less than 4,000 g (20).

Incidences of biochemical pregnancy, clinical pregnancy, ongoing pregnancy, and live birth were calculated using the number of cycles with the above-mentioned outcomes divided by the total number of embryo transfer cycles. Incidences of preterm birth, LBW, and macrosomia was calculated using the number of neonates with these outcomes divided by the total number of live birth babies according to the single or twin births.

### 2.4 Covariate assessment

Based on existing literature (10, 15, 16), the covariates considered in this study for pregnancy outcomes included freeze-all cycle (yes/no), maternal age at oocytes retrieval (in years), maternal age at embryo transfer (in years), maternal body mass index (BMI, in kg/m2 or classified as <18.5/18.5–23.9/24.0–27.9/≥ 28.0 kg/m^2^) (21), gravidity (0/≥1), parity (0/≥1), history of pregnancy loss (0/≥1), infertility duration (in years), etiological factors of infertility (pelvic-tubal factors/ovulation disorders or low ovarian reserve/endometriosis or uterine factors/male factors/other factors), ovarian stimulation protocol [gonadotropin-releasing hormone (GnRH) agonist or antagonist/others], fertilization method (IVF/ICSI), endometrial preparation (artificial cycle with GnRH agonist/artificial cycle/natural cycle/ovarian stimulating cycle), endometrial thickness (in mm or classified as < 10.4/≥ 10.4 mm, 10.4 was the median), number of embryos transferred (1/2), embryo development stage (Day 3/Day 5), and transferred embryo quality (Not good quality/Good quality). For pregnancy outcomes, maternal health status including gestational diabetes mellitus (yes/no), gestational hypertensive disorders (yes/no), gestational thyroid disorders (yes/no), and weight gain during pregnancy (in kg), as well as the sex of the neonate (boy/girl) were additionally regarded as the covariates. Covariate measurements are described in the **Supplementary Material**.

### 2.5 Sample size

The sample size was calculated based on the primary outcome (clinical pregnancy) of the study. According to the previous data from this center, the clinical pregnancy rate of unvaccinated women per FET cycle was 58%. Thus, after assuming an 8% change in this rate among the vaccinated women, and using a two-sided significance level of 5% and a power of 80%, the minimum estimated sample size was 1,218 women. Our study included 2,733 women in the final analysis, which met the sample size requirement.

### 2.6 Statistical analysis

All analyses were performed using SAS version 9.4 (SAS Institute, Cary, NC, USA). All statistical tests were two-sided, and statistical significance was set at P < 0.05. The normally and non-normally distributed continuous variables are expressed as mean ± SD or median (Q1, Q3), respectively, and categorical variables are described as numbers (proportions). Comparisons between groups were accomplished using Mann–Whitney U tests for non-normally distributed continuous variables and χ^2^ tests or Fisher’s exact tests for categorical variables.

In the association analyses, we first evaluated the association between COVID-19 vaccination status and outcomes, followed by an assessment of the impact of vaccination timing. We then estimated the relationships between the time interval and main outcomes based on different vaccination timings. To further explore heterogeneity, we conducted subgroup analyses to estimate the associations between vaccination status and the main outcomes according to multiple maternal or treatment characteristics, including maternal age at oocyte retrieval, maternal age at embryo transfer, maternal BMI, gravidity, parity, history of pregnancy loss, infertility duration, etiological factors of infertility, ovarian stimulation protocol, fertilization method, freeze-all cycles, endometrial preparation, endometrial thickness, number of embryos transferred, embryo development stage, and transferred embryo quality. Interaction analyses were subsequently applied to assess the modifying effect of the characteristics on the associations. Additionally, to rule out the potential confounding by vaccination dose, sensitivity analyses were performed by excluding women who received only one dose of the vaccine.

The associations between COVID-19 vaccination status/timing/time interval and fertility outcomes or pregnancy outcomes in single live birth cycles were evaluated using generalized linear models (GLMs), and such the associations for pregnancy outcomes of twin live births were estimated using generalized estimating equation (GEE) models, with cycles as the random subject. In the GLM or GEE models, binomial distribution and log link function or normal distribution and identity link function were respectively used for estimating the RR for binary outcomes and mean difference for continuous outcomes and their corresponding 95% CIs. A Poisson regression model was used when the log-binomial model failed to converge. Analyses of the interactions between vaccination status and maternal characteristics on outcomes were undertaken by adding an interaction term for vaccination groups and maternal characteristics in the models for each outcome.

Unadjusted and adjusted models were established for each outcome. For fertility outcomes, the adjusted model was adjusted for the propensity score estimated using logistic regression based on the covariates, including freeze-all cycle, maternal age at oocyte retrieval, maternal age at embryo transfer, maternal BMI, gravidity, parity, history of pregnancy loss, infertility duration, etiological factors of infertility, ovarian stimulation protocol, fertilization method, endometrial preparation, endometrial thickness, number of embryos transferred, embryo development stage, and transferred embryo quality. For pregnancy outcomes, the adjusted model was additionally adjusted for gestational complications, weight gain during pregnancy, and the sex of the neonate.

## 3 Results

### 3.1 Participants for final analyses

**Figure 1:**
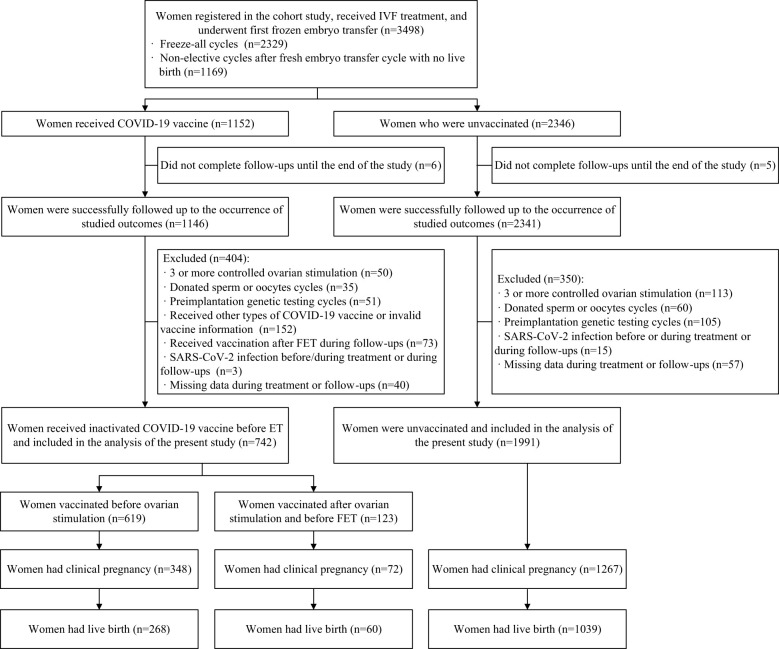
Study flowchart.

A total of 3,498 women who registered for the cohort study, received IVF treatment, and underwent their first FET cycle were included. Among them, after excluding women who did not complete follow-ups until the end of the study (n=11), those who had three or more COS cycles (n=163), those who used donated sperm or oocytes (n=95), those who underwent preimplantation genetic testing cycles (n=165), those who received other types of COVID-19 vaccine or had invalid vaccine information (n=152), those who were vaccinated after FET during follow-ups (n=73), those who had a SARS-CoV-2 infection before/during treatment or during follow-ups (n=18), or those who had missing data during treatment or follow-ups (n=97), 2,733 eligible women (742 in the vaccinated group and 1,991 in the unvaccinated group) were included in the analysis of fertility outcomes. Of these, 1,367 women (328 in the vaccinated group and 1,039 in the unvaccinated group) had live births and were further included in the analysis of pregnancy outcomes (**Figure 1**).

### 3.2 Comparisons of characteristics during IVF treatment and pregnancy between vaccination groups

The vaccination rate among the women before FET was 27.1%. As shown in **Table 1**, the majority of the women had received two doses of the inactive COVID-19 vaccine (88.8%) and had their first dose before OS (83.4%). Compared to the unvaccinated group, the women in the vaccinated group had slightly higher median ages at OS (31.6 vs 31.5 years) and at FET (32.0 vs 31.7 years), and there was a higher proportion of women who were multipara (20.9% vs 15.9%) and those who received ET on Day 3 (26.4% vs 22.3%). Other reproductive and treatment factors were balanced between the two vaccination groups.

**Table 1:** Comparisons of characteristics between COVID-19 vaccination groups among patients undergoing FET.

Characteristic	Vaccinated group (N=742)	Unvaccinated group (N=1991)	*P* ^a^
Maternal vaccination time, n (%)			–
Before OS	619 (83.4)	–	
After OS and before FET	123 (16.6)	–	
Maternal vaccination dose, n (%)			
1	83 (11.2)		
2	659 (88.8)		
Maternal age at oocyte retrieval (yrs.), median (Q1, Q3)	31.6 (29.3, 34.7)	31.5 (29.1, 34.1)	**0.037**
Maternal age at FET (yrs.), median (Q1, Q3)	32.0 (29.6, 35.0)	31.7 (29.3, 34.4)	**0.018**
Maternal BMI (kg/m^2^), median (Q1, Q3)	22.2 (20.2, 24.8)	22.2 (20.3, 24.8)	0.548
Gravidity, n (%)			0.302
0	370 (49.9)	1037 (52.1)	
≥ 1	372 (50.1)	954 (47.9)	
Parity, n (%)			**0.002**
0	587 (79.1)	1675 (84.1)	
≥ 1	155 (20.9)	316 (15.9)	
History of pregnancy loss, n (%)			0.533
0	418 (56.3)	1148 (57.7)	
≥ 1	324 (43.7)	843 (42.3)	
Infertility duration (yrs.), median (Q1, Q3)	3.0 (2.0, 5.0)	3.0 (2.0, 5.0)	0.050
Etiological factors of infertility, n (%)			0.112
Pelvic-tubal factors	471 (63.5)	1257 (63.1)	
Ovulation disorders or low ovarian reserve	89 (12.0)	267 (13.4)	
Endometriosis or uterine factors	74 (10.0)	145 (7.3)	
Male factors	81 (10.9)	226 (11.4)	
Other factors	27 (3.6)	96 (4.8)	
Ovarian stimulation protocol, n (%)			0.172
GnRH agonist or antagonist	669 (90.2)	1828 (91.8)	
Others	73 (9.8)	163 (8.2)	
Fertilization metdod, n (%)			0.873
IVF	595 (80.2)	1602 (80.5)	
ICSI	147 (19.8)	389 (19.5)	
Freeze-all FET cycles, n (%)			0.282
Yes	473 (63.8)	1313 (66.0)	
No	269 (36.2)	678 (34.0)	
Endometrial preparation, n (%) ^b^			0.065
Artificial cycle with GnRH agonist	123 (16.6)	342 (17.2)	
Artificial cycle	377 (50.9)	980 (49.2)	
Natural cycle	181 (24.4)	441 (22.2)	
Ovarian stimulating cycle	60 (8.1)	227 (11.4)	
Endometrial thickness (mm), median (Q1, Q3) ^b^	10.3 (9.3, 11.6)	10.4 (9.3, 11.5)	0.736
No. of embryos transferred, n (%)			0.712
1	615 (82.9)	1662 (83.5)	
2	127 (17.1)	329 (16.5)	
Embryo development stage, n (%)			**0.022**
Day 3	196 (26.4)	443 (22.3)	
Day 5	546 (73.6)	1548 (77.7)	
Transferred embryo quality, n (%)			0.255
Non-good quality	287 (38.7)	723 (36.3)	
Good quality	455 (61.3)	1268 (63.7)	

FET, frozen-thawed embryo transfer; OS, ovarian stimulation; BMI, body mass index; GnRH, gonadotropin-releasing hormone; IVF, *in vitro* fertilization; ICSI, intracytoplasmic sperm injection.

a Comparisons between groups were accomplished using *Mann–Whitney U* tests for continuous variables and *χ* ^2^ tests for categorical variables.

b Endometrial preparation and endometrial thickness were missing for 2 and 3 participants, respectively.

Bold text refers to *p* < 0.05.

As displayed in **Supplementary Table 1**, among the patients who had live births following FET, the incidence of gestational diabetes mellitus was lower in the vaccinated group (10.7% vs. 16.4%), while other pregnancy characteristics were balanced between the two groups.

### 3.3 Associations between COVID-19 vaccination status/timing and fertility and pregnancy outcomes following FET

As presented in **Table 2**, overall, the incidences of fertility outcomes were lower in the vaccinated group compared to the unvaccinated group. After adjusting for potential confounders, compared with the unvaccinated group, the vaccinated group had significantly lower probabilities of clinical pregnancy (RR: 0.92, 95% CI: 0.86, 0.98), ongoing pregnancy (RR: 0.89, 95% CI: 0.82, 0.97), and live birth (RR: 0.89, 95% CI: 0.82, 0.98).

**Table 2:** Associations between COVID-19 vaccination status and fertility/pregnancy outcomes following FET.

Outcome	Vaccination groups		Unadjusted model RR (95% CI)	Adjusted model ^c^ RR (95% CI)
	Vaccinated group	Unvaccinated group		
Fertility outcomes ^a^, n (%)				
Biochemical pregnancy	461 (62.1)	1352 (67.9)	**0.91 (0.86, 0.98)**	0.94 (0.89, 1.00)
Clinical pregnancy	420 (56.6)	1267 (63.6)	**0.89 (0.83, 0.96)**	**0.92 (0.86, 0.98)**
Ongoing pregnancy	341 (46.0)	1076 (54.0)	**0.85 (0.78, 0.93)**	**0.89 (0.82, 0.97)**
Live birth	328 (44.2)	1039 (52.2)	**0.85 (0.78, 0.93)**	**0.89 (0.82, 0.98)**
Pregnancy outcomes				
Single birth ^a^				
Number of babies, n (%)	311	966		
Birth weight, mean ± SD	3323.0 ± 527.5	3340.0 ± 520.0	-17.0 (-83.6, 49.6)	-24.6 (-93.3, 44.1)
Gestational age, mean ± SD	38.8 ± 1.9	38.9 ± 1.8	-0.16 (-0.39, 0.07)	-0.17 (-0.40, 0.07)
Preterm birth, n (%)	30 (9.6)	78 (8.1)	1.19 (0.78, 1.82)	1.27 (0.82, 1.96)
Low birth weight, n (%)	16 (5.1)	49 (5.1)	1.01 (0.58, 1.78)	1.10 (0.62, 1.97)
Macrosomia	29 (9.3)	90 (9.3)	1.00 (0.66, 1.52)	1.02 (0.66, 1.57)
Twin births ^b^				
Number of babies, n (%)	34	146		
Birth weight, mean ± SD	2480.3 ± 444.9	2407.6 ± 392.3	72.7 (-145.6, 291.1)	129.0 (-149.3, 407.2)
Gestational age, mean ± SD	35.9 ± 1.6	35.8 ± 1.8	0.18 (-0.67, 1.04)	0.37 (-0.39, 1.12)
Preterm birth, n (%)	26 (76.5)	104 (71.2)	1.07 (0.79, 1.45)	1.06 (0.64, 1.76)
Low birth weight, n (%)	14 (41.2)	81 (55.5)	0.74 (0.44, 1.25)	0.75 (0.39, 1.42)
Macrosomia, n (%)	0	0	–	–

FET, frozen-thawed embryo transfer; SD, standard deviation; RR, relative risk; CI, confidence interval.

a A generalized linear model was used to estimate the RR (95% CIs).

b A generalized estimating equation model with cycles as the random subject was used to estimate the RR (95% CIs).

c The model was adjusted for the propensity score that was calculated based on the covariates including freeze-all cycles, maternal age at oocyte retrieval, maternal age at embryo transfer, maternal BMI, gravidity, parity, history of pregnancy loss, infertility duration, etiological factors of infertility, ovarian stimulation protocol, fertilization method, endometrial preparation, endometrial thickness, number of embryos transferred, embryo development stage, and transferred embryo quality. For pregnancy outcomes, the model was additionally adjusted for gestational diabetes mellitus, gestational hypertensive disorders, gestational thyroid disorders, weight gain during pregnancy, and the gender of the neonate.

Bold text refers to *p* < 0.05.

As shown in **Table 3**, women who had a COVID-19 vaccination before OS were significantly associated with decreased probabilities of clinical pregnancy (RR: 0.91, 95% CI: 0.84, 0.98), ongoing pregnancy (RR: 0.87, 95% CI: 0.79, 0.96), and live birth (RR: 0.86, 95% CI: 0.78, 0.95). The effect size was similar but did not reach statistical significance in the women vaccinated after OS and before FET (clinical pregnancy: adjusted RR: 0.92, 95% CI: 0.79, 1.07; live birth: adjusted RR: 0.93, 95% CI: 0.78, 1.12).

**Table 3:** Associations between COVID-19 vaccination timing and fertility outcomes following FET.

Fertility outcome	Vaccination timing	n (%)	Unadjusted RR (95% CI)	Adjusted ^b^ RR (95% CI)
Biochemical pregnancy	Unvaccinated	1352 (67.9)	Ref.	Ref.
	Before OS	384 (62.0)	**0.91 (0.85, 0.98)**	0.94 (0.88, 1.01)
	After OS and before FET	77 (62.6)	0.92 (0.80, 1.06)	0.91 (0.79, 1.05)
Clinical pregnancy	Unvaccinated	1267 (63.6)	Ref.	Ref.
	Before OS	348 (56.2)	**0.88 (0.82, 0.95)**	**0.91 (0.84, 0.98)**
	After OS and before FET	72 (58.5)	0.92 (0.79, 1.07)	0.92 (0.79, 1.07)
Ongoing pregnancy	Unvaccinated	1076 (54.0)	Ref.	Ref.
	Before OS	281 (45.4)	**0.84 (0.76, 0.92)**	**0.87 (0.79, 0.96)**
	After OS and before FET	60 (48.8)	0.90 (0.75, 1.09)	0.90 (0.75, 1.07)
Live birth	Unvaccinated	1039 (52.2)	Ref.	Ref.
	Before OS	268 (43.3)	**0.83 (0.75, 0.92)**	**0.86 (0.78, 0.95)**
	After OS and before FET	60 (48.8)	0.93 (0.78, 1.13)	0.93 (0.78, 1.12)

FET, frozen-thawed embryo transfer; OS, ovarian stimulation; RR, relative risk; CI, confidence interval.

a A generalized linear model was used to estimate the RR (95% CIs).

b The model was adjusted for covariates including freeze-all cycles, maternal age at oocyte retrieval, maternal age at embryo transfer, maternal BMI, gravidity, parity, history of pregnancy loss, infertility duration, etiological factors of infertility, ovarian stimulation protocol, fertilization method, endometrial preparation, endometrial thickness, number of embryos transferred, embryo development stage, and transferred embryo quality.

Bold text refers to *p* < 0.05.

As presented in **Table 4**, compared to the unvaccinated group, the women with a ≤ 90-day time interval between vaccination and OS had significantly lower probabilities of clinical pregnancy (RR: 0.86, 95% CI: 0.75, 0.99) and live birth (RR: 0.83, 95% CI: 0.69, 0.99). However, the associations were non-significant in those with a >90-day time interval between vaccination and OS, and in the subgroups of women who were vaccinated after OS and before FET. When comparing outcomes between the >90-day and ≤90-day time intervals, women with a >90-day time interval between vaccination and OS had higher chances of clinical pregnancy and live birth, but the effect size did not reach statistical significance.

**Table 4:** Associations between time interval of COVID-19 vaccination to OS or FET and primary outcomes following FET.

Primary outcome	Vaccination time interval	n (%)	Unadjusted RR (95% CI)	Adjusted RR (95% CI) ^b^	Adjusted RR (95% CI) ^c^
Clinical pregnancy	Unvaccinated	1267 (63.6)	Ref.	Ref.	–
	Vaccinated before OS				
	≤90 days before OS	89 (54.6)	0.86 (0.69, 1.06)	**0.87 (0.76, 0.99)**	Ref.
	>90 days before OS	259 (56.8)	0.89 (0.78, 1.02)	0.94 (0.87, 1.02)	1.09 (0.94, 1.26)
	Vaccinated after OS and before FET				
	≤90 days before FET	27 (57.5)	0.90 (0.62, 1.32)	0.91 (0.71, 1.17)	Ref.
	>90 days before FET	31 (59.2)	0.93 (0.69, 1.25)	1.08 (0.90, 1.29)	1.06 (0.76, 1.48)
Live birth	Unvaccinated	1039 (52.2)	Ref.	Ref.	–
	Vaccinated before OS				
	≤90 days before OS	69 (42.3)	0.81 (0.64, 1.04)	**0.83 (0.69, 0.99)**	Ref.
	>90 days before OS	200 (43.9)	0.84 (0.72, 0.98)	0.90 (0.81, 1.00)	1.07 (0.87, 1.31)
	Vaccinated after OS and before FET				
	≤90 days before FET	22 (46.8)	0.90 (0.59, 1.37)	0.93 (0.69, 1.27)	Ref.
	>90 days before FET	38 (50.0)	0.96 (0.69, 1.32)	1.18 (0.94, 1.48)	1.19 (0.80, 1.76)

FET, frozen-thawed embryo transfer; OS, ovarian stimulation; RR, relative risk; CI, confidence interval.

a A generalized linear model was used to estimate the RR (95% CIs).

b The model was adjusted for the propensity score that was calculated based on the covariates including freeze-all cycle, maternal age at oocyte retrieval, maternal age at embryo transfer, maternal BMI, gravidity, parity, history of pregnancy loss, infertility duration, etiological factors of infertility, ovarian stimulation protocol, fertilization method, endometrial preparation, endometrial thickness, number of embryos transferred, embryo development stage, and transferred embryo quality.

c Comparing the outcomes between the groups with time intervals of ≤90 days or >90 days among women vaccinated before OS or women vaccinated after OS and before FET, respectively.

Bold text refers to *p* < 0.05.

Similar results were observed in the sensitivity analyses conducted after excluding women who received only one dose of the vaccine (**Supplementary Tables 2, 3**).

### 3.4 Subgroup analyses and interaction analyses between COVID-19 vaccination and the primary outcomes following FET by maternal characteristics

Compared with the unvaccinated group, the incidences of clinical pregnancy and live birth were lower in the vaccinated group across most subgroups stratified by maternal and treatment characteristics (**Table 5**). No interaction effect was observed between these characteristics and vaccination status on primary outcomes.

**Table 5:** Subgroup analyses and interaction analyses of the association between COVID-19 vaccination and primary outcomes after FET by maternal characteristics^a^.

Maternal characteristic	Clinical pregnancy				Live birth			
	Vaccinated group	Unvaccinated group	Adjusted ^b^ RR (95% CI)	*P*_for interaction_ ^c^	Vaccinated group	Unvaccinated group	Adjusted ^b^ RR (95% CI)	*P*_for interaction_ ^c^
Maternal age at oocyte retrieval, yrs				0.613				0.967
<35	349 (60.7)	1082 (67.5)	0.93 (0.86, 0.99)		282 (49.0)	915 (57.1)	0.89 (0.81, 0.98)	
≥35	71 (42.5)	185 (47.7)	0.95 (0.78, 1.15)		47 (28.1)	124 (32.0)	0.98 (0.74, 1.28)	
Maternal age at embryo transfer, yrs				0.762				0.729
<35	335 (61.0)	1065 (67.9)	0.92 (0.86, 0.99)		275 (50.1)	902 (57.5)	0.90 (0.82, 0.99)	
≥35	85 (44.0)	202 (47.8)	0.91 (0.77, 1.09)		54 (28.0)	137 (32.4)	0.88 (0.68, 1.14)	
Maternal BMI, kg/m^2^				0.859				0.706
<18.5	30 (49.2)	87 (59.6)	0.95 (0.81, 1.10)		24 (39.3)	73 (50.0)	0.84 (0.60, 1.18)	
18.5–23.9	251 (57.0)	772 (63.1)	0.95 (0.87, 1.04)		194 (44.1)	651 (53.2)	0.90 (0.80, 1.01)	
24.0–27.9	109 (56.8)	294 (65.8)	0.90 (0.79, 1.03)		88 (45.8)	236 (52.8)	0.91 (0.76, 1.08)	
≥28.0	30 (61.2)	114 (65.1)	1.01 (0.76, 1.34)		23 (46.9)	79 (45.1)	1.08 (0.76, 1.52)	
Gravidity, n (%)				0.423				0.212
0	230 (62.2)	693 (66.8)	0.97 (0.90, 1.05)		191 (51.6)	580 (55.9)	0.95 (0.85, 1.06)	
≥1	190 (51.1)	574 (60.2)	0.91 (0.81, 1.01)		138 (37.1)	459 (48.1)	0.85 (0.73, 0.98)	
Parity, n (%)				0.922				0.833
0	347 (59.1)	1104 (65.9)	0.93 (0.86, 1.00)		278 (47.4)	922 (55.0)	0.90 (0.82, 0.99)	
≥1	73 (47.1)	163 (51.6)	0.98 (0.87, 1.12)		51 (32.9)	117 (37.0)	0.99 (0.80, 1.21)	
History of pregnancy loss, n (%)				0.353				0.170
0	256 (61.2)	758 (66.0)	0.97 (0.90, 1.05)		210 (50.2)	631 (55.0)	0.95 (0.86, 1.06)	
≥1	164 (50.6)	509 (60.4)	0.89 (0.79, 1.01)		119 (36.7)	408 (48.4)	0.83 (0.71, 0.97)	
Infertility duration, yrs				0.843				0.782
<2	94 (51.9)	251 (59.9)	0.90 (0.78, 1.05)		74 (40.9)	196 (46.8)	0.91 (0.75, 1.10)	
2–3.9	167 (59.4)	516 (65.1)	0.96 (0.88, 1.04)		134 (47.7)	431 (54.4)	0.91 (0.80, 1.05)	
≥ 4	159 (56.8)	500 (64.2)	0.96 (0.88, 1.05)		121 (43.2)	412 (52.9)	0.92 (0.80, 1.05)	
Etiological factors of infertility, n (%)				0.605				0.429
Pelvic-tubal factor	266 (56.5)	801 (63.7)	0.93 (0.86, 1.01)		205 (43.5)	655 (52.1)	0.90 (0.80, 1.01)	
Ovulation disorders or low ovarian reserve	45 (50.6)	156 (58.4)	0.95 (0.83, 1.09)		36 (40.4)	129 (48.3)	0.86 (0.68, 1.09)	
Other factors	109 (59.9)	310 (66.4)	0.98 (0.85, 1.12)		88 (48.4)	255 (54.6)	0.99 (0.83, 1.17)	
Ovarian stimulation protocol, n (%)				0.612				0.906
GnRH agonist or antagonist	386 (57.7)	1193 (65.3)	0.93 (0.86, 0.99)		305 (45.6)	982 (53.7)	0.91 (0.83, 0.99)	
Others	34 (46.6)	74 (45.4)	1.02 (0.84, 1.23)		24 (32.9)	57 (35.0)	0.96 (0.71, 1.31)	
Fertilization method, n (%)				0.110				0.202
IVF	332 (55.8)	1026 (64.0)	0.94 (0.88, 1.00)		261 (43.9)	839 (52.4)	0.90 (0.81, 0.99)	
ICSI	88 (59.9)	241 (62.0)	1.06 (0.90, 1.24)		68 (46.3)	200 (51.4)	1.06 (0.87, 1.29)	
Freeze-all FET cycles, n (%)				0.619				0.718
Yes	278 (58.8)	879 (67.0)	0.92 (0.85, 1.00)		223 (47.2)	725 (55.2)	0.90 (0.81, 0.99)	
No	142 (52.8)	388 (57.2)	0.92 (0.81, 1.04)		106 (39.4)	314 (46.3)	0.89 (0.77, 1.03)	
Endometrial preparation, n (%)				0.670				0.729
Artificial cycle with GnRH agonist	64 (52.0)	211 (61.7)	0.92 (0.78, 1.08)		54 (43.9)	166 (48.5)	0.98 (0.80, 1.21)	
Artificial cycle	210 (55.7)	622 (63.5)	0.93 (0.84, 1.02)		161 (42.7)	502 (51.2)	0.89 (0.79, 1.01)	
Natural or ovulation- stimulating cycle	145 (60.2)	434 (65.0)	0.96 (0.86, 1.08)		113 (46.9)	371 (55.5)	0.92 (0.79, 1.07)	
Endometrial thickness, mm				0.131				0.249
< 10.4 ^d^	192 (50.9)	607 (61.4)	0.87 (0.79, 0.97)		147 (39.0)	490 (49.5)	0.85 (0.74, 0.97)	
≥ 10.4	228 (62.5)	660 (65.9)	0.97 (0.89, 1.06)		182 (49.9)	549 (54.8)	0.94 (0.84, 1.05)	
No. of embryos transferred, n (%)				0.085				0.185
1	359 (58.4)	1065 (64.1)	0.96 (0.89, 1.03)		280 (45.5)	873 (52.5)	0.94 (0.85, 1.03)	
2	61 (48.0)	202 (61.4)	0.85 (0.70, 1.04)		49 (38.6)	166 (50.5)	0.84 (0.67, 1.05)	
Embryo development stage, n (%)				0.479				0.376
Day 3	92 (46.9)	230 (51.9)	0.91 (0.77, 1.07)		70 (35.7)	184 (41.5)	0.89 (0.72, 1.09)	
Day 5	328 (60.1)	1037 (67.0)	0.94 (0.87, 1.01)		259 (47.4)	855 (55.2)	0.92 (0.83, 1.01)	
Transferred embryo quality, n (%)				0.737				0.971
Non-good quality	134 (46.7)	368 (50.9)	0.97 (0.84, 1.11)		98 (34.1)	283 (39.1)	0.92 (0.77, 1.11)	
Good quality	286 (62.9)	899 (70.9)	0.94 (0.88, 1.00)		231 (50.8)	756 (59.6)	0.93 (0.86, 1.01)	

FET, frozen-thawed embryo transfer; BMI, body mass index; GnRH, gonadotropin-releasing hormone; IVF, *in vitro* fertilization; ICSI, intracytoplasmic sperm injection; RR, relative risk; CI, confidence interval.

a A generalized linear model was used to estimate the RR (95% CIs).

b The model was adjusted for the propensity score that was calculated based on the covariates including freeze-all cycles, maternal age at oocyte retrieval, maternal age at embryo transfer, maternal BMI, gravidity, parity, history of pregnancy loss, infertility duration, etiological factors of infertility, ovarian stimulation protocol, fertilization method, endometrial preparation, endometrial thickness, no. of embryos transferred, embryo development stage, and transferred embryo quality, except for the variable stratified for the subgroup analysis.

c The *P*-values for the interaction were evaluated by adding an interaction term of vaccination and maternal characteristic in the models for each outcome.

d 10.4 mm was the median endometrial thickness.

## 4 Discussion

In the studied population, only 27.1% of women received an inactivated COVID-19 vaccination. We observed reduced clinical pregnancy and live birth rates among the vaccinated women, with no association found with further pregnancy outcomes. The impact of vaccination may vary depending on the timing of the vaccination and the time interval between the vaccination and OS. Significant adverse effects were observed in women who received a vaccination before OS, especially in those with a ≤90-day time interval between the vaccination and OS.

Our findings suggested that COVID-19 vaccination may influence fertility outcomes following IVF-FET without affecting further pregnancy outcomes. The impact of vaccination appears to vary with vaccination timing, particularly posing a higher risk for treatment failure when administered before OS. While no previous evidence has reported the impact of vaccination before OS on outcomes following FET, existing studies have shown conflicting results regarding such associations for fresh ET (14, 18, 22). Wu et al. found no adverse impact on oocyte and embryo quality, ongoing pregnancy rate, or clinical pregnancy rate (10), whereas Shi et al. reported reduced ongoing pregnancy rates when vaccinated within 60 days before fertilization treatment (15). These inconsistencies highlight the need for further investigation into the effect of vaccination before OS on IVF outcomes.

Additionally, this study found lower rates of fertility outcomes among women who received an inactivated COVID-19 vaccination after OS and before FET, compared to unvaccinated women. However, these results were not statistically significant, and the effect was weaker than that observed for vaccination before OS. These findings contrast with studies by Huang et al. and Cao et al., which reported comparable fertility outcomes following FET in the vaccinated and unvaccinated groups (9, 16). The small sample size of women vaccinated after OS and before FET in the present study may have limited the statistical power to detect significant differences. Therefore, studies with larger sample sizes are suggested to verify these results.

Previous research on fresh ET has reported the negative impact of inactivated COVID-19 vaccination on oocyte maturation and fertilization rates (14). This may also explain why the adverse impact of vaccination on the FET outcomes was more pronounced when women were vaccinated before OS in our study. The precise immunological and endocrinological mechanisms underlying these effects remain poorly understood. SARS-CoV-2 infection is known to induce pro-inflammatory responses, leading to cytokine storm and systemic inflammation (23). These responses can elevate the concentration of sex hormones and decrease levels of ovarian reserve indicators, ultimately affecting ovarian follicles and their function (24, 25). Similarly, previous studies have demonstrated that inactivated COVID-19 vaccination can elicit inflammatory responses, albeit weaker ones than those seen with SARS-CoV-2 infection, characterized by the production of cytokines such as IL-1β, IL-6, TNF-α, and TGF-β1, which play key roles in both immune responses and folliculogenesis (26, 27). A recent animal study suggested that both mRNA and inactivated COVID-19 vaccines may trigger systemic inflammatory responses, leading to increased cytokine production. These may detrimentally impact ovarian reserve in rats, primarily through accelerated follicular loss and alterations in apoptotic pathways during folliculogenesis (27). Given these findings, it is plausible that similar inflammatory responses in humans could interfere with folliculogenesis, potentially leading to abnormal oocytes and impaired fertilization and ultimately influencing pregnancy success. This mechanism involving ovarian function may underlie the observed negative impacts on both fresh ET and FET outcomes. Further research is needed to fully elucidate these mechanisms and their impact on reproductive outcomes.

The present study found that the adverse impact of vaccination was mainly observed in women with a time interval of ≤90 days between their vaccination and OS, but not in those with an interval of >90 days or in the time interval subgroups among women vaccinated after OS and before FET. These findings may indicate that to reduce the impact of vaccination on fertility outcomes following IVF-FET, treatment should be initiated at least 90 days after vaccination. The findings regarding women vaccinated after OS and before FET were consistent with previous studies (5, 9). Further studies are needed to verify the results concerning the optimal time interval between vaccination and OS.

In addition, while we found that live birth rates were reduced among vaccinated women, further pregnancy outcomes such as preterm birth and birth weight did not show significant differences. This aligns with limited existing studies that have also reported no significant adverse effects of COVID-19 vaccination on immediate neonatal health indicators (9, 28). However, given the recent introduction of COVID-19 vaccines, the literature on their long-term health impacts is still limited. Further follow-up is needed, and longitudinal studies are essential to assess potential latent health issues over time.

Our study indicates that COVID-19 vaccination may reduce fertility outcomes, specifically clinical pregnancy and live birth chances by 8% and 11%, respectively, among women undergoing IVF-FET, with no impact on further pregnancy outcomes. Although these differences may seem modest, they are clinically meaningful given the inherently low success rates of ART. The absolute differences in rates, which were 7% and 8% for clinical pregnancy and live birth, respectively, correspond to a considerable number of potential pregnancies and live births that could be affected. Notably, vaccination before OS had more pronounced effects. However, vaccination administered more than 90 days prior to ovarian stimulation may help mitigate these potential adverse effects. These findings highlight the importance of vaccination timing and interval for optimizing IVF treatment schedules. They also emphasize the need for informed decision-making and comprehensive counseling to support individualized fertility treatment plans. This research fills critical knowledge gaps regarding the impact of inactivated COVID-19 vaccines on IVF outcomes and offers valuable insights to improve treatment success for vaccinated women.

This is the first relatively large prospective cohort study investigating the associations between inactivated COVID-19 vaccination and fertility and pregnancy outcomes among women undergoing their first FET cycle. Notably, the study takes the lead in evaluating the impact of vaccination before OS on FET outcomes. We conducted a comprehensive analysis of a wide array of fertility and pregnancy outcomes, thereby offering a holistic perspective on the impact of COVID-19 vaccination on FET outcomes. Furthermore, we meticulously estimated the associations while accounting for a broad range of potential confounders, thereby minimizing the influence of confounding effects. We also employed stratified analyses, interaction analyses, and sensitivity analyses to ensure the robustness and reliability of our results. However, several limitations should be addressed. First, this was a single-center study with participants from China, restricting the generalizability of the findings to other populations. Second, this study specifically examined the impact of inactivated COVID-19 vaccines. Given that other vaccine types, such as mRNA or viral vector vaccines, have different compositions and immune mechanisms, our findings cannot be generalized to other types of vaccines. Third, the small sample size for women vaccinated after OS and before FET raised the risk of type II errors. Studies with a sufficient sample size are suggested to verify these results. Finally, although we carried out the analysis by controlling for the potential confounders as much as possible to avoid bias, there were still some unobserved or unknown confounders, for example, some socioeconomic factors, preexisting health conditions, healthcare access, or genetic factors (29), that we could not thoroughly investigate and control. Further prospective studies are needed to verify our findings, and to clarify the optimal time interval between vaccination and OS for women undergoing IVF-FET. Additionally, more basic studies are suggested to explore the underlying mechanisms.

In conclusion, in this prospective cohort study involving women undergoing IVF-FET treatment, inactivated COVID-19 vaccination may be associated with a modest reduction in the chances of clinical pregnancy and live birth without affecting further pregnancy outcomes. Women who received a vaccination before OS may be affected more. However, a vaccination administered more than 90 days prior to ovarian stimulation may help mitigate these potential adverse effects. It is recommended to consider an individual’s vaccination status and timing when formulating a treatment schedule. Further studies are suggested to verify our findings and to elucidate the underlying mechanisms.

## Data Availability

The raw data supporting the conclusions of this article will be made available by the authors, without undue reservation.
