# Supplementary material for: Associations between inactivated COVID-19 vaccination status and timing and fertility and pregnancy outcomes following frozen-thawed embryo transfer: a prospective cohort study
Source: Front Endocrinol (Lausanne). 2025 Jun 26;16:1587251. doi: 10.3389/fendo.2025.1587251 (PMC12240746; doi:10.3389/fendo.2025.1587251)
Supplement: Supplementary file 1 [file DataSheet1.docx]

Supplementary Material

# Supplementary Methods

## Ovarian stimulation protocol and embryo vitrification and thawing procedures

The protocol for COS was determined individually according to female age, body mass index (BMI), basal follicle stimulating hormone (FSH) and antral follicle count (AFC). 89.5% of the studied population received gonadotropin-releasing hormone (GnRH) agonist protocol or GnRH antagonist protocol followed by IVF or ICSI. For women with diminished ovarian reserve, the mild stimulation protocol or luteal phase ovarian stimulation or natural cycle was used. Fertilization was performed using either conventional IVF or ICSI whilst incubated in fertilization media (Vitrolife, Sweden). Embryos were frozen with vitrification following COS and fertilization with either IVF or ICSI. The vitrification and thawing process were done according to standard protocols, as previously described (1). Tools and solutions required for vitrification were obtained from Kitazato (Kitazato BioPharma Co, Japan). The thawed embryos were observed after warming and again before transfer to assess for morphological survival. Embryos with ≥50% intact blastomeres and no signs of fracture of the zona pellucida were defined as surviving.

## Endometrial preparation protocols and frozen-thawed embryo transfer

The type of endometrial preparation was determined according to the experience of the physician based on patients’ characteristics, including natural cycle (NC), ovarian stimulation cycle, hormone replacement therapy (HRT) cycle, and GnRH agonist combined with HRT (GnRH agonist-HRT) cycle. In natural cycles, natural ovulation was monitored with ultrasonography. In ovarian stimulation cycle, human menopausal gonadotropin (HMG, Lizhu Pharmaceuticals, Zhuhai, China) or letrozole combined with HMG was commonly used. Follicle development and endometrial growth were monitored with transvaginal sonography. FET was performed 3 days (cleavage-stage embryos) or 5 days (blastocysts) after ovulation. In HRT cycles, estradiol valerate (Progynova, 4–8mg/day, Bayer, Germany) was started since Days 5 of menstruation. Approximately 10 to 12 days later, 60 mg/day of natural progesterone in oil (Xianju, Zhejiang, China) was injected intramuscularly to prepare the endometrium as soon as the endometrial thickness reached 7 mm and the serum progesterone level was < 1.5 ng/mL. FET was then performed after 4 days (cleavage-stage embryos) or 6 days (blastocysts) of progesterone therapy. In GnRH agonist-HRT cycle, GnRH agonist (Triptorelin, 3.75 mg, Ipsen, France) was administrated on Day 2 or Day 3 of menstrual cycle. Estradiol valerate was provided 28 days later for endometrial proliferation.

The morphologies of embryos were re-evaluated 2–4 h after warming. A good-quality Day 3 embryo was defined as having 7 to 10 cells, 15% or less fragmentation, and smooth blastomeres as well as the absence of multinucleation and vacuolation. A good-quality Day 5 blastocyst was generally defined as a blastocyst of stage 3 or above in the Gardner scoring criteria, with the inner cell mass and trophectoderm scores not including a C grade. Day 5 blastocyst transfer was routinely done for patients who had more than 2 good quality embryos on Day 3; otherwise, Day 3 embryos were transferred. One or two embryos were transferred to endometrium.

## Covariate measurements

Maternal age at oocytes retrieval / embryo transfer were respectively calculated according to birth date and the date of oocytes retrieval / embryo transfer. Height was measured to the nearest 0.1 cm with a stadiometer, and weight was measured to the nearest 0.1 kg with an electronic scale. BMI at enrollment was calculated as weight at enrollment in kilograms divided by height in meters squared, and was classified according to the standards recommended in the “Guidelines for prevention and control of overweight and obesity in Chinese adults” (2). Endometrial thickness (in mm) was estimated using ultrasound scans on the day of progesterone administration, and it categorized as < 10.4 and ≥ 10.4 mm based on the median value.

**References**

1. Shi W, Xue X, Zhang S, Zhao W, Liu S, Zhou H, et al. Perinatal and neonatal outcomes of 494 babies delivered from 972 vitrified embryo transfers. *Fertil Steril*. (2012) 97(6):1338-42. doi: 10.1016/j.fertnstert.2012.02.051

2. Wang, Y.; Sun, M.; Xue, H.; Zhao, W.; Yang, X.; Zhu, X.; Zhao, L.; Yang, Y. Understanding the China Blue Paper on Obesity Prevention and Control and policy implications and recommendations for obesity prevention and control in China. *Zhonghua Yu Fang Yi Xue Za Zhi*. (2019) 53:875–84.

# Supplementary Tables

**Supplemental Table 1.** Comparisons of characteristics during pregnancy and birth between COVID-19 vaccination groups among patients give live births after FET

| Characteristics | Vaccinated group | Unvaccinated group | *P* ^a^ |
| --- | --- | --- | --- |
| Patients give live births after FET, n | 328 | 1039 |  |
| Gestational diabetes mellitus, n (%) | 35 (10.7) | 170 (16.4) | **0.012** |
| Gestational hypertensive disorders, n (%) | 31 (9.5) | 117 (11.3) | 0.358 |
| Gestational thyroid disorders, n (%) | 5 (1.5) | 22 (2.1) | 0.501 |
| Weight gain during pregnancy, median (Q1, Q3) | 14.0 (10.0, 16.0) | 14.0 (10.0, 16.0) | 0.456 |
| No. of births |  |  | 0.241 |
| 1 | 311 (94.8) | 966 (93.0) |  |
| 2 | 17 (5.2) | 73 (7.0) |  |
| Neonatal sex, boy, n (%) | 191 (58.2) | 558 (53.7) | 0.151 |
| FET, frozen-thawed embryo transfer.  ^a^ Comparisons between groups were accomplished using *Mann-Whitney U* tests for continuous variables and *χ*^2^ tests for categorical variables.  Bold text refers to *p* < 0.05. | | | |

**Supplemental Table 2.** Sensitivity analysis: association between COVID-19 vaccination and fertility outcomes after FET ^a^

| Outcomes  n (%) | Vaccination groups | | Unadjusted model  RR (95% CI) ^b^ | Adjusted model ^c^  RR (95% CI) |
| --- | --- | --- | --- | --- |
|  | Unvaccinated  group | Vaccinated  group |  |  |
| Biochemical pregnancy | 1352 (67.9) | 409 (62.1) | 0.91 (0.85, 0.98) | 0.95 (0.89, 1.01) |
| Clinical pregnancy | 1267 (63.6) | 370 (56.2) | 0.88 (0.82, 0.95) | 0.92 (0.85, 0.99) |
| Ongoing pregnancy | 1076 (54.0) | 298 (45.2) | 0.84 (0.76, 0.92) | 0.88 (0.81, 0.97) |
| Live birth | 1039 (52.2) | 287 (43.6) | 0.83 (0.76, 0.92) | 0.88 (0.80, 0.97) |
| FET, frozen-thawed embryo transfer; SD, standard deviation; RR, relative risk; CI, confident interval.  ^a^ Excluding women with 1 dose vaccination. N=2650.  ^b^ Generalized linear model was used to estimate the RR (95% CIs).  ^c^ Model was adjusted for the propensity score that was calculated based on the covariates including freeze-all cycle, maternal age at oocytes retrieval, maternal age at embryo transfer, maternal BMI, gravidity, parity, history of pregnancy loss, infertility duration, etiological factors of infertility, ovarian stimulation protocol, fertilization method, endometrial preparation, endometrial thickness, number of embryos transferred, embryo development stage, and transferred embryo quality.  Bold text refers to *p* < 0.05. | | | | |

**Supplemental Table 3.** Sensitivity analysis: association between COVID-19 vaccination timing and fertility outcomes after FET ^a^

| Pregnancy outcomes | Vaccination timing | n (%) | Unadjusted  RR (95% CI) ^b^ | Adjusted ^c^  RR (95% CI) |
| --- | --- | --- | --- | --- |
| Biochemical pregnancy | Unvaccinated | 1352 (67.9) | Ref. | Ref. |
|  | Before OS | 343 (61.9) | **0.91 (0.82, 0.98)** | 0.95 (0.88, 1.02) |
|  | After OS and before FET | 66 (62.9) | 0.93 (0.80, 1.08) | 0.91 (0.79, 1.06) |
| Clinical pregnancy | Unvaccinated | 1267 (63.6) | Ref. | Ref. |
|  | Before OS | 309 (55.8) | **0.88 (0.81, 0.95)** | **0.90 (0.83, 0.98)** |
|  | After OS and before FET | 61 (58.1) | 0.91 (0.77, 1.08) | 0.92 (0.78, 1.08) |
| Ongoing pregnancy | Unvaccinated | 1076 (54.0) | Ref. | Ref. |
|  | Before OS | 248 (44.8) | **0.83 (0.75, 0.92)** | **0.86 (0.78, 0.95)** |
|  | After OS and before FET | 50 (47.6) | 0.88 (0.72, 1.08) | 0.89 (0.72, 1.07) |
| Live birth | Unvaccinated | 1039 (52.2) | Ref. | Ref. |
|  | Before OS | 237 (42.8) | **0.82 (0.74, 0.91)** | **0.85 (0.77, 0.95)** |
|  | After OS and before FET | 50 (47.6) | 0.91 (0.74, 1.12) | 0.91 (0.75, 1.11) |
| FET, frozen-thawed embryo transfer; OS, ovarian stimulation; RR, relative risk; CI, confident interval.  ^a^ Excluding women with 1 dose vaccination. N=2650.  ^b^ Generalized linear model was used to estimate the RR (95% CIs).  ^c^ Model was adjusted for covariates including freeze-all cycle, maternal age at oocytes retrieval, maternal age at embryo transfer, maternal BMI, gravidity, parity, history of pregnancy loss, infertility duration, etiological factors of infertility, ovarian stimulation protocol, fertilization method, endometrial preparation, endometrial thickness, number of embryos transferred, embryo development stage, and transferred embryo quality.  Bold text refers to *p* < 0.05. | | | | |
